# Egg white hydrolysate and peptide reverse insulin resistance associated with tumor necrosis factor-α (TNF-α) stimulated mitogen-activated protein kinase (MAPK) pathway in skeletal muscle cells

**DOI:** 10.1007/s00394-018-1753-7

**Published:** 2018-06-28

**Authors:** Myoungjin Son, Jianping Wu

**Affiliations:** 1 Department of Agricultural, Food and Nutritional Science, University of Alberta, Edmonton, AB T6G 2P5 Canada; 2 Cardiovascular Research Centre, University of Alberta, Edmonton, AB T6G 2P5 Canada

**Keywords:** Insulin resistance, Skeletal muscle cells, Egg white protein hydrolysate, IRW, Insulin signaling

## Abstract

**Purpose:**

Excessive formation of tumor necrosis factor-α (TNF-α), a pro-inflammatory cytokine, has been implicated in the development of insulin resistance in obesity and type-2 diabetes. In skeletal muscle, chronic exposure to TNF-α impairs insulin-stimulated glucose uptake and insulin signaling. The aim of this study is to investigate the effects of enzymatic egg white hydrolysate (EWH) and its responsible peptide, IRW, on TNF-α-induced insulin resistance and the underlying molecular mechanisms using rat skeletal muscle cells (L6 cells).

**Methods:**

Insulin resistance was induced by treating L6 cells with 5 ng/ml TNF-α for 24 h. Effects of EWH and IRW on glucose uptake were detected by glucose uptake assay, glucose transporter 4 (GLUT4) translocation by immunofluorescence, and western blot, while insulin-signaling pathway and mitogen-activated protein kinase (MAPK) pathway were investigated using western blot.

**Results:**

Adding both EWH and IRW significantly improved glucose uptake in TNF-α-treated cells, increased activation of insulin receptor substrate (IRS-1) tyrosine residue and protein kinase B (Akt), whereas decreased activation of IRS-1 serine residue. In addition, TNF-α-induced activation of p38-mitogen-activated protein kinase (p38) and c-Jun N-terminal kinases (JNK) 1/2 were decreased by either EWH or IRW treatment.

**Conclusion:**

EWH and IRW improve impaired insulin sensitivity by down-regulating the activation of p38 and JNK1/2 in TNF-α-treated skeletal muscle cells.

## Introduction

Metabolic syndrome is represented by a group of interrelated disorders, including obesity, hyperglycemia, hyperlipidemia, and hypertension [1]. It is also a significant risk factor for cardiovascular disease and increased morbidity and mortality [2]. The incidence of metabolic syndrome is rapidly increasing worldwide, becoming a major public and clinical problem [3]. Insulin resistance is a major underlying mechanism responsible for metabolic syndrome [4]. Although pancreatic β-cell dysfunction is another feature for development of type 2 diabetes, insulin resistance is regarded as primary factor to initiate the development of hyperglycemia prior to β-cell failure [5, 6]. Since skeletal muscle is the predominant site of insulin-stimulated glucose uptake, numerous recent studies have focused on discovering therapeutic compound to improve insulin resistance in skeletal muscle [7].

In normal skeletal muscle metabolism, insulin stimulates auto-phosphorylation of insulin receptor on tyrosine residues and consequent phosphorylation of insulin receptor substrates 1 and 2 (IRS-1 and IRS-2). This results in the activation of phosphatidylinositol 3-kinase (PI3K) and protein kinase B (Akt) allowing glucose transporter protein 4 (GLUT4) storage vesicles to move to the plasma membrane for glucose uptake [8]. After uptake, glucose is phosphorylated by hexokinase, and then stored as glycogen or enters into mitochondria for glucose oxidation through the tricarboxylic acid (TCA) cycle and electron transport system [9]. In insulin resistance state, insulin signaling is impaired, which results in decreased capacity of insulin to stimulate the translocation of GLUT4, whereas the GLUT4 protein content is unchanged [10].

TNF-α is a pro-inflammatory cytokine that plays a key role in the mediation of immune response as a multi-functional regulator of inflammation, cell apoptosis, cytotoxicity, and production of other cytokines [11]. Multiple studies revealed that excessive TNF-α concentrations have been implicated in the development of insulin resistance in obesity and type 2 diabetes [12–15]. In skeletal muscle, chronic exposure to TNF-α impairs insulin-stimulated glucose uptake and GLUT4 translocation by increasing the phosphorylation of Ser residue of IRS-1 while decreasing the phosphorylation of Akt [16–20]. The molecular mechanism underlying TNF-α-mediated insulin resistance is well recognized. TNF-α-activated oxidative and pro-inflammatory pathways are mediated by p38-mitogen-activated protein kinase (p38), c-Jun N-terminal kinases (JNK), and extracellular signal-regulated protein kinases 1 and 2 (ERK1/2) [16, 17]; inhibitor of p38 completely restored insulin-dependent glucose uptake and insulin-signaling pathway [16]. In addition, TNF-α was shown to suppress insulin sensitivity by the inactivation of the key energy sensor, 5′ adenosine monophosphate-activated protein kinase (AMPK), via the transcriptional upregulation of protein phosphatase 2C (PP2C) [20].

There has been an explosion of scientific research in regard of food-derived bioactive protein peptides [21]. Our previous research has identified three egg white protein ovotransferrin-derived ACE-inhibitory peptides, IRW, IQW, and LKP [22–29]. Among them, IRW showed anti-inflammatory and anti-oxidant ability in vitro and in vivo [25–29]. Our recent study also further suggested that IRW improved insulin resistance in angiotensin II-treated L6 skeletal muscle cells [30]. While these individual peptides possess strong bioactivities, the commercialization is still limited due to the unavailability of technologies and high cost of purification techniques. Hence, there is a great therapeutic applicability of using enzymatic hydrolysate of whole egg white. Our previous research has shown that egg white hydrolysate (EWH) prepared by thermolysin and pepsin-reduced blood pressure, improved vascular relaxation, and reduced aortic angiotensin II receptors 1 (AT1R) expression in spontaneously hypertensive rats (SHR) [31]. Our recent studies also further suggested that EWH might have a beneficial effect on insulin sensitivity and metabolic syndrome. EWH treatment on 3T3-F442A pre-adipocytes increased the cell differentiation, insulin-signaling pathway, and inhibited inflammatory markers such as cyclooxygenase-2 (COX-2) and JNK [32].

Although commercially produced EWH has been shown to improve glucose homeostasis [33], its underlying mechanism as well as the peptides responsible for the action have not been fully investigated. Herein, we investigated the effect of EWH and egg white-derived peptide, IRW, on TNF-α-induced insulin resistance and its underlying molecular mechanism by testing glucose uptake, GLUT4 translocation, and insulin-signaling pathway using rat skeletal muscle cells (L6). To induce insulin resistance, we incubated L6 myotubes with TNF-α for 24 h. The role of EWH and IRW in preventing TNF-α-induced insulin resistance was studied by co-incubation of TNF-α in the presence of EWH or IRW.

## Materials and methods

### Materials

TNF-α, insulin, dithiothreitol (DTT), and Triton-X-100 were from Sigma-Aldrich (St Louis, MO, USA). Dulbecco’s modified Eagle medium (DMEM), fetal bovine serum (FBS), and antibiotic–antimycotic solution and horse serum were purchased from Gibco/Invitrogen (Carlsbad, CA, USA). Dihydroethidium (DHE), Hoechst 33342, was purchased from Thermo Fisher Scientific (Thermo Fisher Scientific, Burlington, Canada). IRW was synthesized by Genscript (Piscataway, NJ, USA). Peptide sequence and purity (99.8%) were validated by HPLC–MS/MS.

### Antibodies

Rabbit monoclonal primary antibodies against phospho-insulin receptor 1 (Tyr632 and Ser307), and insulin receptor 1 and p38 were obtained from Santa Cruz Biotechnology Inc (SantaCruz, CA, USA). Rabbit monoclonal primary antibody against GLUT4 and mouse monoclonal primary antibody against α-tubulin and phospho-p38 were bought from Abcam (Cambridge, MA, USA). Rabbit monoclonal primary antibody against phospho-Akt (Ser473), Akt was bought from Cell Signaling Technology Inc. (Danvers, MA, USA). Goat anti-rabbit IRDye 680RD secondary antibody or donkey anti-mouse 800CW secondary antibody was purchased from Licor Biosciences (Lincoln, NE, USA). Rabbit monoclonal primary antibody against phospho JNK and mouse monoclonal primary antibody against JNK was purchased from RD system (Minneapolis, MN, USA).

### Preparation of egg white hydrolysate (EWH)

Hydrolysis of egg white was carried out according to our previous method [22]. EWH was desalted with 50% acetonitrile/deionized water using Sep-Pak C18 cartridges (product #: WAT043345, Waters, Ontario, Canada) to remove salts in the hydrolysate for using in cell experiments.

### Cell culture

Rat-derived L6 myoblasts were obtained from American-Type Culture Collection (Manassas, VA; ATCC_ numbers: CRL-1458). The cells were grown in DMEM supplemented with 10% FBS and 1% v/v antibiotic–antimycotic solution (10,000 units/ml penicillin G, 10 mg/ml streptomycin, and 25 mg/ml amphotericin B) at 5% CO_2_ and 37 °C until they reached 80% confluence. For further differentiation, the cells were cultured in DMEM containing 2% horse serum for 6–7 days. The media were changed every 48 h and cells were used at the stage of myotubes (60–70%) when GLUT4 expression is the highest [34].

### Glucose uptake assay

Glucose uptake assay was examined by the procedure described previously [35, 36] with slight modifications. Briefly, L6 myoblasts (5 × 10^4^ cells/well) were subcultured into Nunc 24-place multiwell plates and grown for 9–10 days until they formed myotubes. The myotubes were incubated with 5 mg/ml of EWH or 100 µM of IRW in serum-free DMEM 2 h. Next, 5 ng/ml TNF-α was co-incubated for another 24 h to induce insulin resistance as described previously by Alvaro et al. [16] with a slight modification.

Next, the myotubes were kept for 2 h in Krebs–Henseleit buffer (pH 7.4) containing 0.1% bovine serum albumin (BSA), 10 mM Hepes, and 2 mM sodium pyruvate (KHH buffer). The myotubes were then cultured in KHH buffer containing 11 mM glucose in the absence or the presence of 1 µM of insulin for another 4 h. Glucose concentrations in KHH buffer were determined with Glucose CII-Test kit (Wako Pure Chemical Industries, Ltd., Osaka, Japan) and the amounts of glucose consumed were calculated from the differences in glucose concentrations between before and after culture.

### Insulin-signaling pathway assay

The L6 myotubes were incubated with 5 mg/ml of EWH or 100 µM of IRW in serum-free DMEM for 2 h, followed by treatment with 5 ng/ml TNF-α for different time conditions. To detect the total and phosphorylated proteins of the insulin-signaling pathway, the myotubes were kept for 2 h in KHH buffer (pH 7.4) and then incubated in KHH buffer containing 11 mM glucose in the absence or the presence of 1 µM insulin for 30 min. At the end of incubation, L6 myotubes were lysed in boiling hot Laemmle’s buffer containing 50 mM DTT and 0.2% Triton-X-100 to prepare samples for western blotting. To extract the protein from cell membrane, a Mem-PER™ Plus Membrane Protein Extraction Kit (Thermo Fisher Scientific) was used. Briefly, cells were washed with cell wash solution and centrifuged at 300×*g* for 5 min. After discarding the supernatant, cells were resuspended and incubated with permeabilization buffer at 4 °C. Next, permeabilized cells were centrifuged for 15 min at 16,000×*g*. The supernatant containing cytosolic proteins was transferred into a new tube and the pellet was incubated with solubilization buffer at 4 °C for 30 min. After centrifuging for 15 min at 16,000×*g*, the supernatant containing solubilized membrane and membrane-associated proteins was transferred to a new tube for western blotting analysis. The cell lysates were then run in 9% sodium dodecyl sulfate polyacrylamide gel electrophoresis (SDS-PAGE), transferred to nitrocellulose membranes, and immunoblotted with primary antibodies. After incubating with secondary antibodies, protein bands were detected by Licor Odyssey BioImager (Licor Biosciences, NE, USA) and quantified by densitometry using Image Studio Lite 5.2.

### Immunofluorescence

L6 myotubes were incubated with 5 mg/ml of EWH or 100 µM of IRW 2 h prior to treatment with 5 ng/ml TNF-α for 24 h. The myotubes were then incubated for 2 h in KHH buffer and then kept in KHH buffer containing 11 mM glucose in the absence or the presence of 1 µg of insulin for 30 min. The myotubes were fixed in 3.75% paraformaldehyde. To selectively stain the proteins on cell membranes, the cells were not permeabilized. After washing twice with PBS, cells were blocked with 1% BSA in PBS for 1 h and incubated with anti-GLUT4 primary antibody overnight at 4 °C. After washing three times with PBS, cells were treated with Alexa Fluor 546 conjugated goat anti-rabbit secondary antibody for 1 h at room temperature. The cell nuclei were stained with Hoechst 33342 dye from molecular probes. Finally, after washing three times with PBS, cells were examined with an EVOS FL Auto Cell Imaging System (Thermo Fisher Scientific). All images presented are 100× magnification.

### Statistical analysis

All data are presented as the mean ± SEM. The data were evaluated by IBM SPSS version 22. Differences between the mean values were assessed using one- or two-way ANOVA, followed by Tukey multiple comparisons test if applicable. Statistical significance was considered for values of *p* < 0.05.

## Results

### Effect of EWH or IRW on glucose uptake in L6 myotubes

**Fig. 1:**
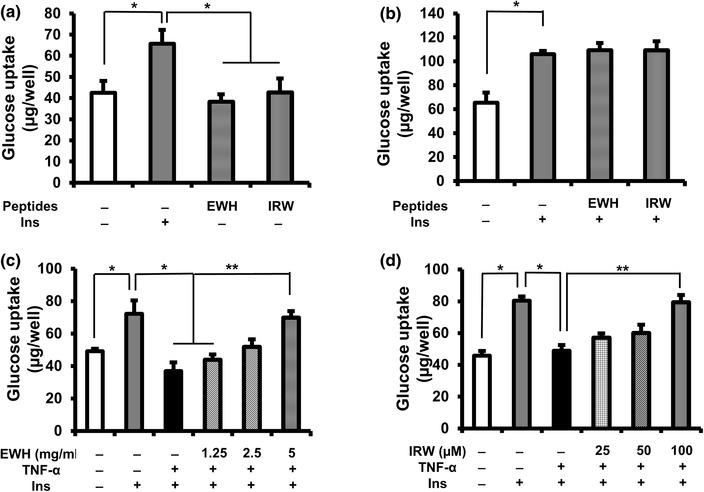
Effects of EWH or IRW on glucose uptake in L6 myotubes. Effects of EWH or IRW on glucose uptake in the absence or the presence of insulin (**a, b**). The myotubes were preincubated in 24-place multiwell plates in Krebs–Henseleit–Hepes buffer (KHH buffer) without glucose for 2 h. They were then incubated in KHH buffer containing 11 mM glucose without or with insulin and 5 mg/ml of EWH or 100 µM of IRW for 4 h. Effects of EWH or IRW on insulin-stimulated glucose uptake in TNF-α-treated L6 myotubes (**c, d**). The myotubes were incubated with EWH (1.25, 2,5, and 5 mg/ml) or IRW (25, 50, and 100 µM) 2 h prior to the treatment of 5 ng/ml of TNF-α for 24 h. Next, the myotubes were kept for 2 h in KHH buffer. The myotubes were then cultured in KHH buffer containing 11 mM glucose in the absence or the presence of 100 nm of insulin for another 4 h, and then, the glucose uptake was measured using a Glucose CII-Test Kit. Each value represents the mean ± SEM of five independent experiments. Single asterisk indicates *p* < 0.05 as compared to insulin alone. Double asterisk indicates *p* < 0.05 as compared to the insulin and TNF-α

To investigate the effect of EWH or IRW on insulin-independent glucose uptake in L6 myotubes, EWH or IRW was incubated with glucose–KHH buffer for 4 h in the absence of insulin. Neither EWH nor IRW stimulated basal glucose uptake (Fig. 1a). To test the possible insulin-sensitizing effect of peptides, EWH or IRW was incubated with insulin for 4 h and no significant changes in glucose uptake were detected in the presence of EWH or IRW (Fig. 1b).

TNF-α has been reported to decrease insulin-stimulated glucose uptake in skeletal muscle [16, 19, 20]. Therefore, we detected whether EWH or IRW can protect the action of insulin from TNF-α treatment. The myotubes were incubated with EWH (1.25, 2.5, and 5 mg/ml) or IRW (25, 50, and 100 µM) for 2 h followed by the co-incubation with TNF-α for 24 h. TNF-α significantly decreased insulin-stimulated glucose uptake by 50% compared to the one with insulin alone (Fig. 1c, d). However, pre-incubation of EWH (5 mg/ml) or IRW (100 µM) prior to TNF-α treatment significantly restored the decreased glucose uptake.

### Effect of EWH or IRW on insulin-signaling pathway in TNF-α-treated L6 myotubes

**Fig. 2:**
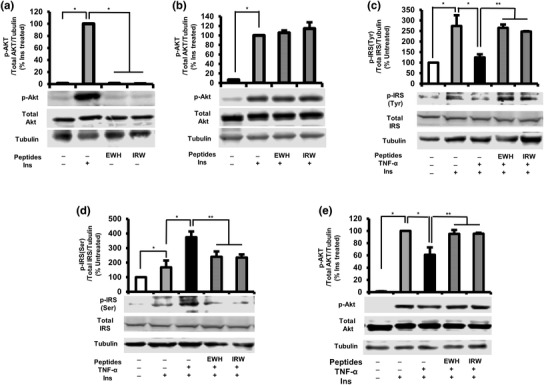
Effects of EWH or IRW on insulin-signaling pathway in L6 myotubes. Effects of EWH or IRW on insulin signaling in the absence or the presence of insulin (**a, b**). The myotubes were treated with 5 mg/ml of EWH or 100 µM of IRW for 2 h followed by treatment with 100 nm of insulin for 30 min. Effects of EWH or IRW on insulin-signaling pathway in TNF-α-treated L6 myotubes (**c, d, e**). The myotubes were treated with 5 mg/ml of EWH or 100 µM of IRW for 2 h followed by treatment with 5 ng/ml of TNF-α for 24 h. L6 myotubes were preincubated in KHH buffer for 2 h. They were then incubated in KHH buffer containing 11 mM glucose without or with 100 nm of insulin for 30 min. The cells were lysed and western blotting of the lysates was performed with antibodies against p-Akt (**a, b, e**), total Akt (**a, b, e**), p-IRS-1 tyr 632 (**c**), p-IRS-1 Ser 307 (**d**), total IRS-1 (**c, d**), and α-tubulin (loading control). A set of representative images is shown. Data are presented as mean ± SEM of three independent experiments. Single asterisk indicates *p* < 0.05 as compared to insulin alone. Double asterisk indicates *p* < 0.05 as compared to insulin and TNF-α

To investigate whether EWH or IRW can stimulate insulin signaling in the absence or the presence of insulin, the phosphorylation of Akt was detected. The treatment of both EWH and IRW did not affect the Akt activation in the absence or the presence of insulin as expected from the results of glucose uptake (Fig. 2a, b).

It is well documented that TNF-α impairs insulin-signaling pathway [16–19]. Consistent with the literatures, our results showed that TNF-α significantly reduced the activation of IRS-1 through increased phosphorylation of serine residue of IRS-1, while decreased phosphorylation of tyrosine residue with respect to the untreated cells (Fig. 2c, d). Moreover, TNF-α resulted in subsequent deactivation of Akt (Fig. 2e). Thus, we next assessed the effect of EWH or IRW on IRS-1 and Akt phosphorylation in TNF-α-treated L6 cells. The treatment of EWH or IRW increased the phosphorylation level of tyrosine residue (Fig. 2c) of IRS-1, whereas the phosphorylation of serine residue was significantly decreased (Fig. 2d) in comparison with TNF-α alone. Furthermore, the phosphorylation of Akt was also significantly restored by either EWH or IRW (Fig. 2e). Taken together, these data demonstrate that normalized insulin-signaling pathway by both EWH and IRW is a contributing factor to the improved glucose uptake in TNF-α-induced insulin resistance.

### Effect of EWH or IRW on GLUT4 translocation in TNF α-treated L6 myotubes

**Fig. 3:**
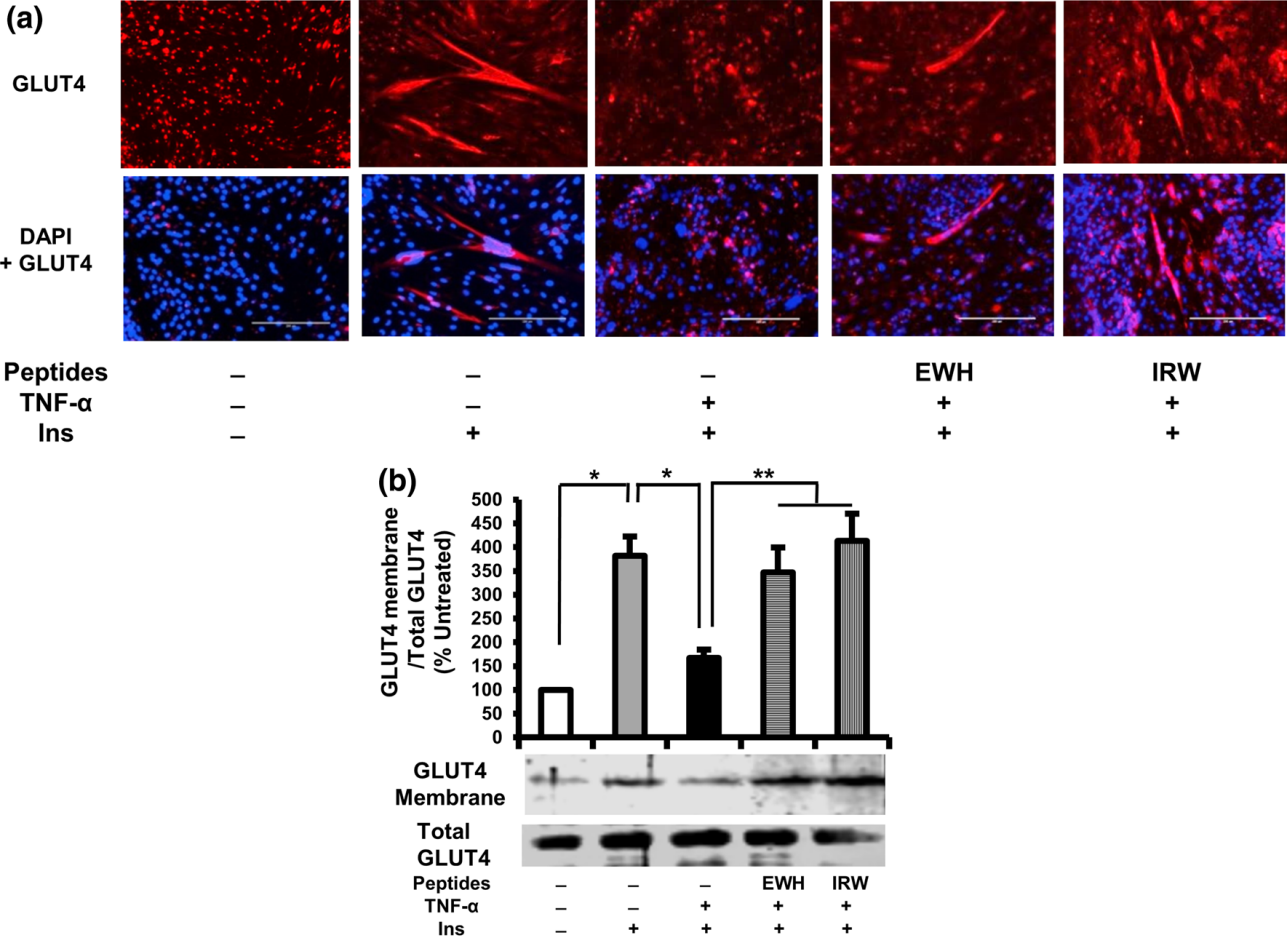
Effects of EWH or IRW on GLUT4 translocation in TNF-α-treated L6 myotubes. The myotubes were treated with 5 mg/ml of EWH or 100 µM of IRW for 2 h followed by treatment with 5 ng/ml of TNF-α for 24 h. L6 myotubes were preincubated in KHH buffer for 2 h. They were then incubated in KHH buffer containing 11 mM glucose without or with 100 nm of Insulin for 30 min (**a**). GLUT4 localization was detected using immunofluorescence technique and western blotting. Cellular localization of GLUT4 proteins is shown in red fluorescence (top) and merged image is also shown (below). A representative set of images from three independent experiments is shown. **b** Membranes were separated from the cells. The expression of GLUT4 was tested by western blot. Each value represents the mean ± SEM of three independent experiments. Single asterisk indicates *p* < 0.05 as compared to insulin alone. Double asterisk indicates *p* < 0.05 as compared to insulin and TNF-α

Having observed that both EWH and IRW improve insulin-stimulated glucose uptake as well as insulin-signaling pathway, we next evaluated the effect of EWH or IRW on GLUT4 translocation in TNF-α-treated L6 cells using two different experiment techniques. First, we detected the GLUT4 abundance in L6 myotubes using immunofluorescence. As shown in Fig. 3a, red fluorescence (top) shows cellular localization of GLUT4, while DAPI + GLUT4 (down) indicates merging of GLUT4 and nuclei. The robust decrease in the GLUT4 level was detected in TNF-α treated cells as compared to insulin-treated one. The treatment of IRW or EWH improved the defects in GLUT4 abundance on cellular membrane in TNF-α-treated cells. This effectiveness of immunofluorescence result was confirmed by western blot (Fig. 3b). The result showed that both EWH and IRW significantly elevated the expression of GLUT4 on plasma membrane in comparison with TNF-α-treated cells, indicating the possibility that both EWH and IRW re-established defective GLUT4 translocation in TNF-α-treated cells.

### Effect of EWH or IRW on p38 MAPK and JNK1/2 activation in TNF-α-treated L6 myotubes

**Fig. 4:**
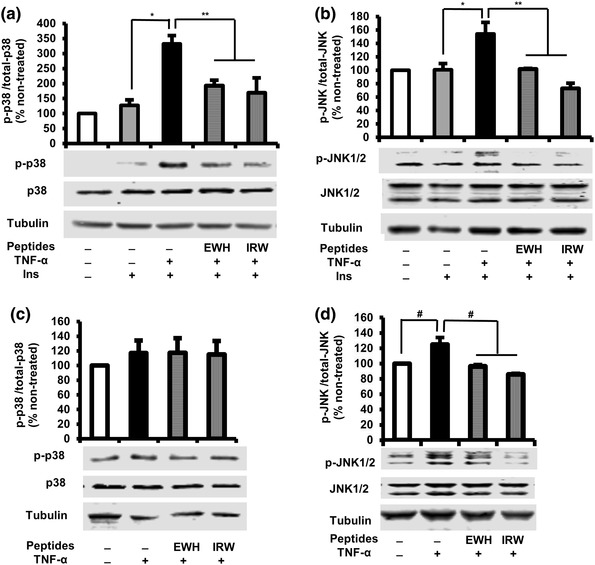
Effects of EWH or IRW on p38 and JNK1/2 activation in TNF-α-treated L6 myotubes. The myotubes were treated with 5 mg/ml of EWH or 100 µM of IRW for 2 h followed by the treatment with 5 ng/ml of TNF-α for 0.5 h. The cells were lysed and western blotting of the lysates was performed with antibodies against p-p38 (**a, c**), p-38 (**a, c**), p-JNK1/2 (**b, d**), JNK1/2 (**b, d**), and α-tubulin (loading control). A set of representative images was shown. Data were presented as mean ± SEM of three independent experiments. Single asterisk indicates *p* < 0.05 as compared to insulin alone. Double asterisk indicates *p* < 0.05 as compared to insulin and TNF-α. ^#^*p* < *0.05* as compared to TNF-α alone

Extensive studies in different diabetic animal models and cells unambiguously showed that TNF-α activated p38 and JNK1/2, resulting in interruption of insulin signaling [16, 17]. We, therefore, sought to examine whether the effect of EWH or IRW is associated with the phosphorylation of p38MAPK and JNK1/2. While TNF-α treatment significantly increased the activation of both p38 and JNK1/2 in the presence of insulin as compared to those in untreated cells (Fig. 4 a, b), only the phosphorylation of JNK1/2 was increased by TNF-α in the absence of insulin (Fig. 4d, e). However, pretreatment with EWH and IRW diminished the activation of p38 (Fig. 4a), as well as JNK1/2 (Fig. 4b, d). These results indicate that the prevention of insulin resistance by EWH and IRW may stem from inhibiting p38 and JNK1/2 activities.

## Discussion

Insulin resistance is one of the main defining clinical features in metabolic syndrome. Given that skeletal muscle is characterized as a major tissue for insulin-mediated glucose disposal, skeletal muscle cell is an appropriate model to explore natural food component with potential application against insulin resistance. In the current study, we showed that both EWH and IRW can improve insulin resistance in TNF-α-treated L6 skeletal muscle cells. TNF-α treatment suppressed insulin-stimulated glucose uptake via the disruption of insulin signaling and GLUT4 translocation, while incubating EWH or IRW 2 h prior to TNF-α significantly restored these defects. The mechanism underlying was potentially through the reduction of phosphorylation of p38 and JNK1/2.

It has become increasingly evident over the last decade that chronic inflammation is major contributor to the development and progression of insulin resistance [37, 38]. The results from the clinical studies have revealed a clear association between the pro-inflammatory signaling pathway and insulin resistance. Elevated levels of pro-inflammatory biomarkers such as TNF-α, interleukin-6 (IL-6) and interleukin-8 (IL-8), and C-reactive protein (CRP) have all been reported in various insulin resistance states [39–43]. Animal and human studies also support the link between TNF-α and insulin resistance [15, 17]. Hotamisligil et al. have reported that obese mice genetically deficient of TNF-α or its receptor ameliorated glucose homeostasis [15]. In addition, phosphorylation of Tyr residue in p-IRS and GLUT4 expression in skeletal muscle was increased compared to obese-TNF+/+ group. TNF-α infusion for 4 h decreased peripheral insulin-mediated glucose uptake, without influencing endogenous gluconeogenesis from the liver [17]. Thus, inhibition of the action of TNF-α contributes to improved insulin sensitivity. Interestingly, in this study, we observed that pre-incubation of EWH or IRW for 2 h recovered TNF-α impaired glucose uptake and GLUT4 abundance on cell membrane. In addition, both EWH and IRW improved IRS-1 and Akt activation, which suggests the possibility in insulin sensitivity enhancement.

The underlying mechanism of TNF-α-induced insulin resistance has been reported to mediate through the inflammatory signaling pathway including the activation of three MAPKs, p38 [16], JNK1/2, and ERK1/2 [17]. It is well recognized that MAPK signaling is required for maintaining normal metabolic condition, but the excessive activation of MAPKs is associated with inflammatory diseases including obesity and diabetes [44].

Over-expressed MKK6/3-p38/MAPK in L6 myotubes was reported to deteriorate the action of GLUT4 via down-regulation of GLUT4 gene expression [45], whereas knock-down MAP4K4, the upstream kinase of ERK1/2 and JNK, inhibits TNF-α-induced insulin resistance [46]. Collectively, these data highlight that increased MAPK activity plays a significant role in the pathogenesis of insulin resistance in skeletal muscle. Our current study showed that TNF-α treatment increased the phosphorylation of p38 and JNK1/2, but significantly attenuated by EWH or IRW further acknowledging the involvement of MAPKs in their underlying mechanism.

IRW was initially identified as an ACE-inhibitory peptide [24]. While it showed anti-hypertensive effect in spontaneously hypertensive rats (SHR), interestingly, it also decreased pro-inflammatory/oxidative stress markers [25]. In particular, IRW exhibited anti-inflammatory and anti-oxidant effect in human umbilical vein endothelial cells (HUVECs) by reduction of TNF-α-induced superoxide generation [27, 28]. Our results suggest the possibility that specific ACE-inhibitory peptides derived from food protein might possess various capacities to expand their application in the disorders associated with oxidative stress and inflammation mechanism.

While EWH and IRW showed similar effect and underlying mechanism in our current study, further investigation in vivo will be required to confirm the efficacy of EWH after the digestion. Furthermore, considering TNF-α is an important contributing factor to the development of insulin resistance, additional studies are warranted to determine the precise role of EWH and IRW in relation with the action of increased circulating TNF-α in insulin resistance animals.

In conclusion, the present study shows, for the first time, that EWH processed by thermolysin and pepsin, and egg white-derived tripeptide, IRW, improves impaired insulin sensitivity by down-regulating the activation of p38 and JNK1/2 in TNF-α-treated skeletal muscle cells. Our results provide an important insight for further investigating of food protein hydrolysates and peptides as a novel therapeutic alternation against metabolic syndrome and help to illustrate their mechanism of action.
